# 

**Published:** 1908-08-22

**Authors:** 


### THE GRADUATES' MEDICAL SCHOOLS.

DIARY FOR WEEK AUGUST 24 to 29.

### THE POST-GRADUATE COLLEGE, West London Hospital, Hammersmith, W.

**At 12 noon.**
**August 24,** Dr. Low, **Pathological Demonstration.**
**At 5 p.m.**
**August 25,** Dr. Abraham, **Cases of Skin Diseases.**
**At 5 p.m.**
**August 26,** Mr. Pardoe, **The Diagnosis of Surgical Diseases of the Kidney.**

---

THE HOSPITAL August 22, 1908.

*Name* ..............................................................

*Address* ..............................................................

..............................................................

This Coupon must accompany manuscript or contributions intended for The Hospital.

